# Supplementary figures and images for: A Genomic Survey of the Natural Product Biosynthetic Potential of Actinomycetes Isolated from New Zealand Lichens
Source: mSystems. 2023 Feb 7;8(2):e01030-22. doi: 10.1128/msystems.01030-22 (PMC10134820; doi:10.1128/msystems.01030-22)

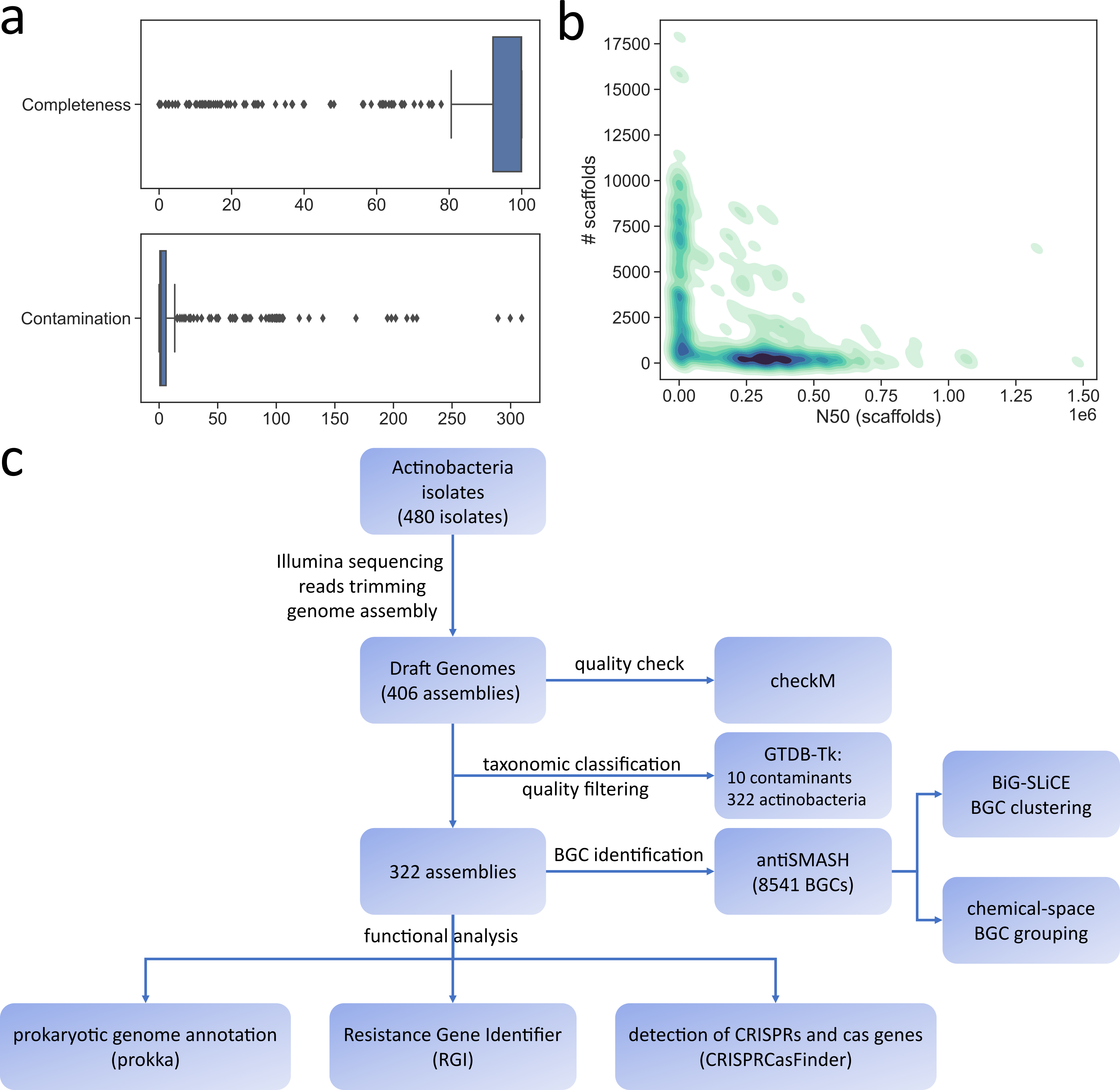

Supplement: FIG S1 [file msystems.01030-22-s0004.tif]

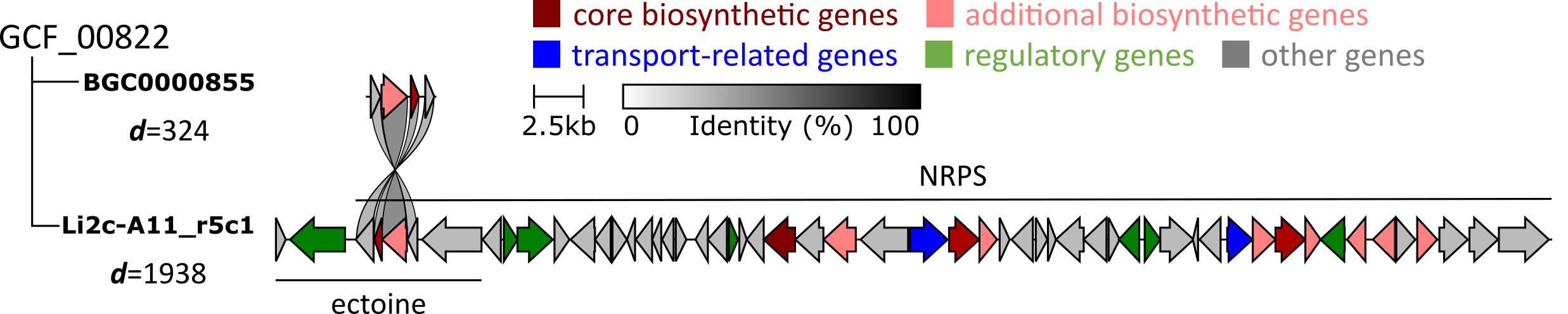

Supplement: FIG S3 [file msystems.01030-22-s0006.tif]
